# Connecting different heart diseases through intercellular communication

**DOI:** 10.1242/bio.058777

**Published:** 2021-09-08

**Authors:** Tania Martins-Marques

**Affiliations:** 1 Univ Coimbra, Coimbra Institute for Clinical and Biomedical Research (iCBR), Faculty of Medicine, 3000-548 Coimbra, Portugal; 2 Univ Coimbra, Center for Innovative Biomedicine and Biotechnology (CIBB), 3004-504 Coimbra, Portugal; 3 Clinical Academic Centre of Coimbra (CACC), 3004-561 Coimbra, Portugal

**Keywords:** Gap junctions, Extracellular vesicles, Tunneling nanotubes, Cell-based therapies, Myocardial infarction, Cardiac hypertrophy

## Abstract

Well-orchestrated intercellular communication networks are pivotal to maintaining cardiac homeostasis and to ensuring adaptative responses and repair after injury. Intracardiac communication is sustained by cell–cell crosstalk, directly via gap junctions (GJ) and tunneling nanotubes (TNT), indirectly through the exchange of soluble factors and extracellular vesicles (EV), and by cell–extracellular matrix (ECM) interactions. GJ-mediated communication between cardiomyocytes and with other cardiac cell types enables electrical impulse propagation, required to sustain synchronized heart beating. In addition, TNT-mediated organelle transfer has been associated with cardioprotection, whilst communication via EV plays diverse pathophysiological roles, being implicated in angiogenesis, inflammation and fibrosis. Connecting various cell populations, the ECM plays important functions not only in maintaining the heart structure, but also acting as a signal transducer for intercellular crosstalk. Although with distinct etiologies and clinical manifestations, intercellular communication derailment has been implicated in several cardiac disorders, including myocardial infarction and hypertrophy, highlighting the importance of a comprehensive and integrated view of complex cell communication networks. In this review, I intend to provide a critical perspective about the main mechanisms contributing to regulate cellular crosstalk in the heart, which may be considered in the development of future therapeutic strategies, using cell-based therapies as a paradigmatic example.

This Review has an associated Future Leader to Watch interview with the author.

## Abstract (precis)

**Summary:** A finely tuned interplay between distinct intercellular communication mechanisms is required for both homeostasis and remodeling of the diseased heart, posing as relevant target to improve the development of therapeutic strategies.

## Introduction

Cardiovascular diseases are the leading cause of global mortality. Among the most frequent disorders are coronary artery disease, very often preceding acute myocardial infarction (MI), and systemic arterial hypertension and aortic stenosis, usually resulting in pressure overload and cardiac hypertrophy (reviewed in Heusch, 2020 and Nakamura and Sadoshima, 2018).

Both ischemia and pressure overload are key initiators of adaptive responses involving cellular and extracellular compartments of the heart, which contribute to changes in the shape, mass and volume of ventricles, collectively known as cardiac remodeling (Heusch, 2020; Nakamura and Sadoshima, 2018). The remodeling process is associated with alterations in electric and mechanical properties of cardiomyocytes, but also involves other cardiac-resident cells, including endothelial cells, smooth muscle cells, fibroblasts and immune cells (Pinto et al., 2016). Hence, a well-orchestrated cell–cell and cell–extracellular matrix (ECM) crosstalk is vital to drive coherent responses after injury, as well as for the maintenance of cardiac homeostasis.

**Fig. 1.**
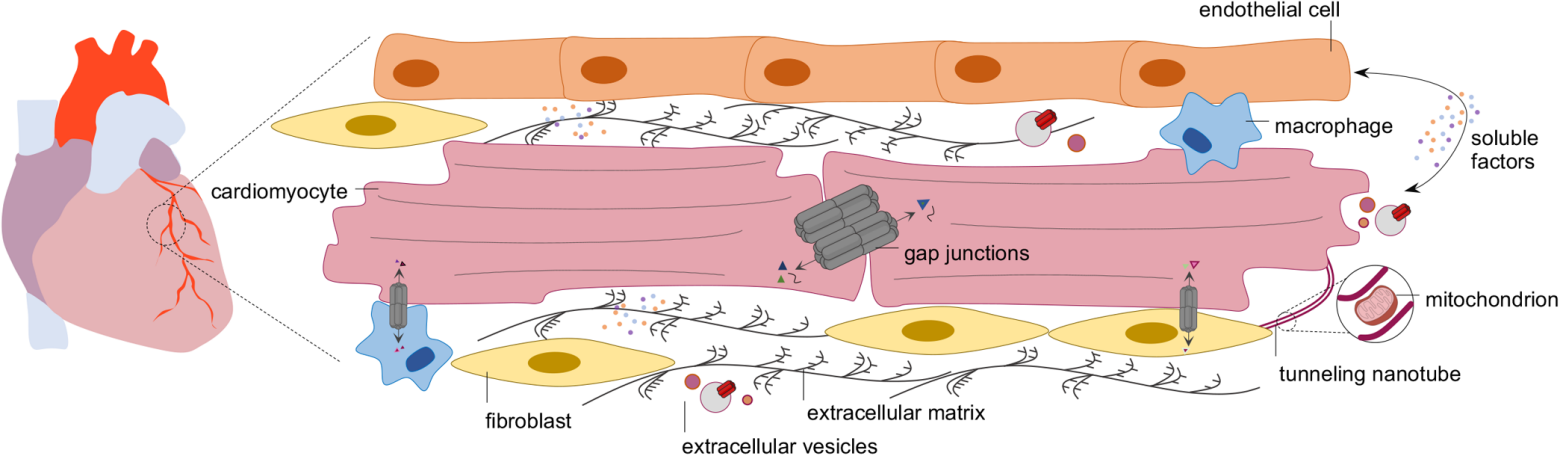
**Distinct intercellular communication mechanisms in the heart.** Transfer of electric and metabolic signals between cardiac cells can occur directly through gap junctions (GJ) and tunneling nanotubes (TNT). GJ connect the cytoplasm of adjacent cells, enabling the transfer of molecules less than 1 kDa (e.g. ions, inositol triphosphate, glucose and cyclic AMP), while TNT allow the exchange of larger biomolecules, including organelles. At longer distances, secretion of soluble cytokines, growth factors, or extracellular vesicles can modify the behavior of target cells. Connecting various cardiac cell populations, the extracellular matrix provides structural support, acts as reservoir of multiple bioactive factors, and participates on signal transduction, thus regulating intercellular communication.

Although working in an integrated and coordinated manner, intercellular communication mechanisms, including gap junctions (GJ), tunneling nanotubes (TNT), extracellular vesicles (EV), and cell–matrix interactions have been often approached as distinct and independent entities. Grounded on the solid knowledge gathered over recent years, I will provide a comprehensive view of different intercellular crosstalk mechanisms in cardiac health and disease (Fig. 1), which may inspire the establishment of more effective therapeutic strategies. Particularly, cell-based therapies which emerged as powerful approaches to promote cardiac repair following injury, will be discussed as a paradigmatic example that would significantly benefit from an holistic understanding of cell–cell crosstalk mechanisms (Tompkins et al., 2017).

## Direct cell–cell communication mechanisms

### Gap junctions

Formed after the docking of two hemichannels on juxtaposed cells, GJ constitute an intercellular conduit for the exchange of ions and small molecules with less than 1 kDa (reviewed in Ribeiro-Rodrigues et al., 2017a). GJ are predominantly localized at the longitudinal termini of cardiomyocytes, within intercalated discs, enabling the unidirectional propagation of electrical impulses, which sustains the coordinated contraction of the cardiac muscle (Smyth et al., 2010). In addition, GJ ensure electrical coupling between cardiac fibroblasts and cardiomyocytes (Vasquez et al., 2010), as well as between macrophages and cardiomyocytes (Hulsmans et al., 2017).

Each hemichannel is constituted by six transmembrane proteins termed connexins (Cx). In contrast with GJ, hemichannels are usually closed in basal conditions (Contreras et al., 2003). However, metabolic inhibition, low extracellular or high intracellular calcium (Ca^2+^) levels can trigger hemichannel opening, thereby allowing communication between the cytoplasm and the extracellular milieu (Lissoni et al., 2021), which has been associated with arrhythmogenesis in heart failure (HF) (De Smet et al., 2021). Although the human genome encodes for 21 different connexins, working cardiomyocytes and the cardiac conduction system mainly express Cx43, Cx40 and Cx45 (Verheule and Kaese, 2013). Cx26 is also expressed in human atrial cardiomyocytes, with its presence being detected in cytoplasmic vesicles, mitochondria and myofibrils, but not at the intercalated discs (Moscato et al., 2018). Interestingly, expression of cardiac Cx30.3 can be induced in animal models of heart disease and was detected in hypertrophic cardiomyopathy in human patients (Okamoto et al., 2020). Since most of the currently available evidence spans from studies on Cx43, in the context of this work, I will mainly discuss the role of cardiac Cx43.

Beyond its classical role as a GJ channel-forming protein, the presence of Cx43 was also detected in mitochondria and EV, namely in cardiac cells (Martins-Marques et al., 2020a; Rodriguez-Sinovas et al., 2005). Although the exact functions of non-junctional Cx43 remain poorly established, mitochondrial Cx43 was reported to impact on respiration, potassium influx and oxygen consumption (Boengler et al., 2012), while Cx43 hemichannels at the EV surface were proposed as a novel mechanism to facilitate EV cargo delivery into target cells (Martins-Marques et al., 2016; Soares et al., 2015).

### Tunneling nanotubes

The transfer of molecules between connected cells can also occur via thin (20–700 nm) and long (up to 100 µm in length) F-actin-rich membranous structures, termed TNT, which allow the exchange of ions, proteins, organelles and pathogens (reviewed in Cordero Cervantes and Zurzolo, 2021). Ample evidence has demonstrated the involvement of TNT in the diffusion of inositol triphosphate (IP3), mediating the spread of Ca^2+^ signals (Lock et al., 2016), as well as in the stimulation of cell proliferation (Vallabhaneni et al., 2012) and cell death (Arkwright et al., 2010). Importantly, the expression of Cx43 in both coupled cells is required for TNT-mediated electrical coupling, suggesting a relevant interplay between GJ and TNT (Lock et al., 2016; Wang et al., 2010). Despite bona fide TNT being exclusively constituted by actin, tubulin-containing TNT (>0.7 µm in diameter) have also been uncovered in various cell types, including cardiac myoblasts (He et al., 2010). *In vitro* evidence revealed that TNT connecting cardiomyocytes and fibroblasts enable the transfer of lysosomes [26], mitochondria and calcium (Ca^2+^) (He et al., 2011). Although the biological relevance of such connections remains poorly established, TNT-mediated mitochondrial transfer from cardiac fibroblasts was shown to rescue cardiomyocytes from apoptosis (Shen et al., 2018).

### Cell- and vesicle-ECM interactions

Mainly synthesized by fibroblasts, the cardiac ECM is a highly organized fiber meshwork, composed by collagens, elastin, proteoglycans and glycoproteins, which not only provides structural support to the various cell populations of the heart, but also act as a reservoir of growth factors, cytokines and EV, actively modulating cell–cell communication (Zhang et al., 2019). Despite being frequently viewed as a mechanism to regulate their bioavailability, binding of growth factors to ECM components can also regulate cellular responses. Accordingly, interaction between vascular endothelial growth factor (VEGF) and fibronectin synergistically modulate proliferation and migration of endothelial cells (Wijelath et al., 2006), which may have important implications for angiogenesis.

Non-structural roles of the ECM mostly rely on matricellular proteins, such as thrombospondins, secreted protein acidic and rich in cysteine (SPARC) and tenascin-C that contribute to cell adhesion, motility and mechanotransduction (Imanaka-Yoshida and Aoki, 2014), as well as on cell–matrix interactions, mainly involving integrins at the cell surface (Zhang et al., 2019). In agreement, fibronectin and collagen secretion by embryonic cardiac fibroblasts is able to activate β1 integrin signaling and promote cardiomyocyte proliferation (Ieda et al., 2009), ascribing crucial functions for the ECM as a hub for cardiac cell–cell crosstalk, which should be exploited in future therapeutic approaches.

ECM is a highly dynamic structure that can be modified by the proteolytic action of matrix metalloproteinases (MMPs), as well as by non-enzymatic glycation, ultimately affecting its functions (Spinale et al., 2013). A fine-tuned regulation of synthesis, deposition and degradation of ECM components is vital for development of the heart and valves, which can be altered during cardiomyocyte differentiation and in response to pathological stimuli (Lincoln et al., 2006). For example, qualitative and quantitative differences in the synthesis of hyaluronan and proteoglycans during cardiomyocyte differentiation was observed (Chan et al., 2010), which may be relevant to inspire and improve the design of cell-based therapies (Silva et al., 2016), discussed below.

## Long-distance communication mechanisms

### Soluble factors

Secretion of soluble mediators is crucial to ensure cell–cell crosstalk in the heart, displaying important functions in cardiac development and cardiomyocyte function, immunomodulation, scar formation and fibrosis (reviewed in Fountoulaki and Dagres, 2015). Myriad cardioactive factors have been reported, including nitric oxide (NO), angiotensin II (Ang II), endothelin-1 (ET-1), VEGF, natriuretic peptides, neuregulin, ROS, transforming growth factor β (TGFβ), TNFα and interleukin (IL)-6, which can either act in an autocrine or paracrine manner (Basma et al., 2019; Iyer et al., 2012; Schultz et al., 2002). Since some of these signals can be originated by various cardiac-resident cell populations, *in vitro* studies are crucial to better characterize their cellular sources, as well as their biological effects in specific target cells. In agreement, cardiac fibroblast-conditioned medium induced hypertrophy of cardiomyocytes, which was associated with the presence of high levels of several cytokines, including VEGF, TNFα, IL-6 and IL-17 (LaFramboise et al., 2007).

One of the best-known paracrine modulators in the healthy myocardium is NO, produced mainly by endothelial cells, but also by cardiomyocytes, which plays pivotal roles in the regulation of vascular tone, cardiomyocyte contractility and in cardioprotection (Fountoulaki and Dagres, 2015). In agreement, triply deficient nitric oxide synthase (NOS)^−/−^ mice manifest features of metabolic syndrome, display vascular dysfunction and develop spontaneous MI (Nakata et al., 2008). Under pathological conditions, certain soluble factors, such as TGFβ (Schultz et al., 2002) and IL-6 (Fuchs et al., 2003; Lai et al., 2012), become critical players of orchestrated stress responses, contributing for cardiac remodeling following ischemia and pressure overload.

### Extracellular vesicles

EV represent an heterogeneous population of nanosized bilayered vesicles that participate in cell-cell crosstalk at longer distances (reviewed in Mathieu et al., 2019), including intra- and inter-organ communication. Secreted by putatively every cell type in the human body, EV are found in a wide variety of biological fluids, such as the blood, urine and saliva (Théry et al., 2018). EV enclose numerous active biomolecules, such as proteins, nucleic acids and lipids that may determine the biological responses triggered by the vesicles, once taken up by target cells (Haraszti et al., 2016). Besides their involvement in orchestrated intercellular communication, EV can make part of a pro-survival mechanism, by conveying the release of unwanted potentially toxic material, namely protein aggregates or partially depolarized mitochondria, by injured cells (Phinney et al., 2015), and mediating the exchange of molecular chaperones to mount an advantageous organismal proteostasis response (Takeuchi et al., 2015). Successful delivery of EV-enclosed information to distant recipient cells requires the migration of EV through the surrounding ECM, which is determined not only by the mechanical properties of the matrix (Lenzini et al., 2020), but also by the size and surface protein composition of the vesicles (Skliar et al., 2018). More recently, the presence of water channels on EV was demonstrated to contribute for dynamic regulation of their volume, facilitating EV transport across the ECM (Lenzini et al., 2020). Hence, disease-associated edema may interfere with the biophysical properties of EV, ultimately impacting on intercellular communication.

Although with different mechanisms of biogenesis, both exosomes (50–150 nm) and microvesicles (100-1000 nm) participate in the communication between different cardiac cell types (Almeida Paiva et al., 2019; Bang et al., 2014; Ribeiro-Rodrigues et al., 2017b), or between the heart and other organs, such as the bone marrow (Cheng et al., 2019). Despite EV from cardiac origin have been found in the blood stream (Pironti et al., 2015), under physiological conditions, circulating EV predominantly derive from blood and vascular cells. Changes in the content of circulating EV have been associated with various cardiovascular disorders, ascribing to these vesicles an important role as disease biomarkers (Deddens et al., 2016; Ling et al., 2020; Martins-Marques et al., 2020a).

In the heart, myriad EV-mediated communication axis regulate important biological processes, such as angiogenesis, fibrosis, hypertrophy and apoptosis (Martins-Marques et al., 2020b). Thus, cardiomyocyte-derived EV modulate gene expression in fibroblasts (Waldenström et al., 2012), while EV derived from cardiac endothelial cells (Song et al., 2014) and cardiomyocytes (Almeida Paiva et al., 2019) are able to alter the phenotype of immune cells. Given that immune cell function is critical for cardiac immunesurveillance, as well as to sustain inflammatory responses in the diseased myocardium, it would be of utmost importance to better characterize the impact of such EV-mediated cellular phenotypes. Long-distance communication by EV can also be modulated by the action of soluble factors, including TGFβ and platelet-derived growth factor (PDGF), which were demonstrated to affect the mRNA content in cardiomyocyte EV, likely determining distinct responses in target cells (Gennebäck et al., 2013).

## Intercellular communication changes associated with myocardial infarction, hypertrophy and heart failure

Not only pressure and volume stress, mutations in sarcomere-related genes, but also loss of contractile mass due to ischemic cell death in MI, are associated with compensatory hypertrophic growth of the left ventricle (Nakamura and Sadoshima, 2018). Although initially required to preserve cardiac function, long-term activation of cardiac hypertrophy is associated with the development of HF (Schwinger, 2021).

**Fig. 2.**
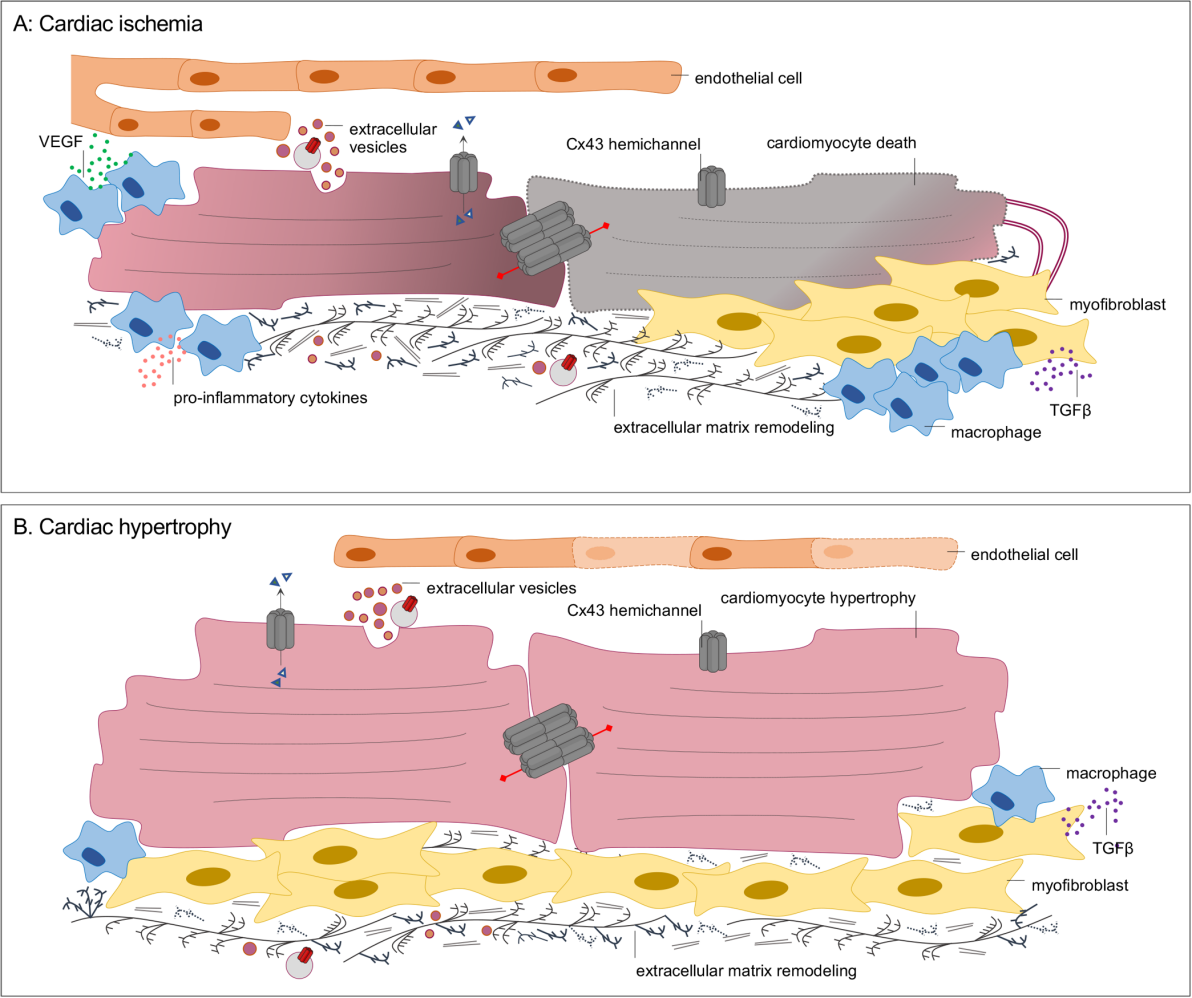
**Cell–cell crosstalk in the injured myocardium.** Both cardiac ischemia (A) and pressure overload-induced hypertrophy (B) have been associated with a downregulation of gap-junction-mediated communication between cardiomyocytes, increased Cx43 hemichannel opening, changes in the content of extracellular vesicles, as well as enhanced secretion of soluble factors, including transforming growth factor β (TGFβ). Besides TGFβ-mediated conversion of fibroblasts into myofibroblasts, dysregulated expression of matrix-degrading enzymes and their tissue inhibitors drive extracellular matrix remodeling. While soluble factors and EV induce angiogenesis during ischemia, capillary rarefaction is usually observed following maladaptive hypertrophy. During ischemia, increased formation of tunneling nanotubes is also observed.

In recent years, multiple unbiased approaches have contributed to establish the signaling pathways and networks involved in cardiac remodeling, which has paved the way to identifying novel disease biomarkers and putative therapeutic targets (Lau et al., 2018). For example, single-cardiomyocyte transcriptomics demonstrated that transition from compensated hypertrophy into HF primarily involves activation of extracellular-signal-regulated protein kinases 1 and 2 (ERK1/2), nuclear respiratory factors 1 and 2 (NRF1/2) and p53 signaling (Nomura et al., 2018). Besides intrinsic morphological and functional cardiomyocyte alterations, crosstalk with other cardiac cell populations and with the ECM is instrumental for pathological remodeling in the setting of MI and pressure overload (Fig. 2). In agreement, transcriptome data was correlated with an upregulation of regulatory genes involved in inflammatory responses and ECM following post-MI healing (Li et al., 2019) and isoproterenol-induced hypertrophy (Lau et al., 2018). A significant dysregulation of long non-coding RNA was found in cardiac fibroblasts upon pressure overload (Piccoli et al., 2017), while distinctive patterns of circulating soluble cytokines (Lebedeva et al., 2020), EV-enclosed proteins and miRNA (Otero-Ortega et al., 2021) were also identified, corroborating the importance of long-distance intra-cardiac and systemic communication in remodeling of the heart.

Multi-omics approaches are now emerging as vital tools to allow a more comprehensive view of the myriad pathways involved in complex cardiac diseases (Lau et al., 2018). Importantly, disease signature tracing should consider the high heterogeneity within the cardiac tissue, which could be leveraged by region- and cell-type specific profiling studies (Doll et al., 2017). In the following sections, I will discuss evidence demonstrating how cell–cell and cell–matrix communication are affected during cardiac disease, as well as the role of cellular crosstalk in remodeling of the injured heart, aiming to find common mechanisms.

## Pathological changes on direct cell–cell communication via gap junctions and tunneling nanotubes

Multiple studies have demonstrated a downregulation of GJIC in cardiomyocytes following MI and pressure overload-induced cardiac hypertrophy (Emdad et al., 2001; Qu et al., 2009), mostly related with changes in the post-translational modifications of Cx43, which strongly impact on channel gating. For example, dysfunctional GJIC and arrhythmogenesis have been associated with dephosphorylation of Ser325/Ser328/Ser330 in mouse models of cardiac hypertrophy and ischemia (Lampe, 2006; Qu et al., 2009, 2011). Conversely, ischemia-induced phosphorylation of Ser368, as well as Cx43 ubiquitination, were implicated in GJ channel internalization, lateralization (Martins-Marques et al., 2015b, 2020c; Smyth et al., 2014) and autophagy degradation of Cx43 during ischemia and reperfusion (Martins-Marques et al., 2015a), contributing to GJIC downregulation. Co-localization between internalized GJ and the autophagosome marker LC3 was also observed in canine failing hearts (Hesketh et al., 2010), suggesting that autophagy degradation of Cx43 represents a general feature during disease-associated cardiac remodeling, likely constituting a putative therapeutic target.

In contrast, an upregulation of Cx43 in fibroblasts is very often observed under the same pathological conditions (Camelliti et al., 2004), contributing to an increased GJIC between injured cardiomyocytes and fibroblasts, which may have important implications upon electrical conduction across the scar tissue (Vasquez et al., 2010). It has been previously demonstrated that the expression of Cx43 can be modulated in response to the ECM composition, likely influencing GJIC and hemichannel function (Guo et al., 2001; Imbeault et al., 2009). Given that ECM remodeling is a major hallmark in both pressure overload-induced cardiac hypertrophy and post-MI repair, altered ECM content may interfere with GJIC. In the opposite direction, the impact of disease-associated accumulation of fibroblast Cx43 on ECM dynamics should not be discarded.

Electrotonic coupling in the cardiac scar border tissue was also suggested to involve the formation of TNT-like structures between myocytes and nonmyocytes (Quinn et al., 2016). Consistent with this hypothesis, recent evidence demonstrated that the number of TNT connecting cardiomyocytes and fibroblasts was significantly increased in ischemic co-cultures *in vitro* (Batista-Almeida et al., 2020). In addition, TNT-mediated communication between cardiomyocytes and fibroblasts was associated with the spread of inflammatory injury following stimulation of the β-adrenergic receptor, a typical hypertrophic stimuli (Shen et al., 2020). However, the functional consequences of these findings *in vivo* remain unknown. As demonstrated by the examples above, bi-directional crosstalk between cardiomyocytes and fibroblasts is vital to maintain electric and metabolic coupling in the diseased heart. Besides GJ- and TNT-mediated direct contacts, conditioned medium obtained from ischemic fibroblasts affected the electrophysiological properties of cardiomyocytes, uncovering an important role for paracrine signals in this process (Vasquez et al., 2010). Nonetheless, the relative contribution of soluble factors and EV remains to be clarified.

## ECM remodeling in cardiac diseases

Although matrix remodeling is a common feature of several cardiovascular disorders, the underlying injury determines the molecular and biochemical alterations on the ECM network (reviewed in Frangogiannis, 2019). Accordingly, MI-induced cardiomyocyte death triggers an acute pro-inflammatory response, accompanied by an upregulation of matrix-degrading enzymes that stimulates early ECM turnover (Lindsey et al., 2006; Santer et al., 2020; Spinale et al., 2010). As macrophages acquire a reparative phenotype, characterized by increased secretion of anti-inflammatory cytokines and growth factors, including TGFβ, cardiac fibroblasts transdifferentiate into myofibroblasts, leading to the formation of a scar (Weber et al., 2013). On the other hand, mechanical and neurohumoral stimulation, in the setting of pressure overload, drive a matrix-synthetic program that initially aims to preserve cardiac output, but eventually decompensates, with a concomitant dysregulated expression between MMPs and their tissue inhibitors (TIMPs) (Nakamura and Sadoshima, 2018).

Regardless of the disease etiology, myofibroblasts are the main effector cells driving ECM remodeling, being characterized by the expression of contractile proteins, increased production of both MMPs and ECM components, namely collagens (Petrov et al., 2002; Weber et al., 2013). In agreement, an upregulation of MMP-9 has been associated with pathological remodeling of the cardiac ECM (Sung et al., 2018, 2020; Tao et al., 2004), contributing not only to exacerbate matrix turnover, but also for inflammation following MI (Deleon-Pennell et al., 2016). In addition, downregulation of MMP-7 improved survival rates of mice following MI (Lindsey et al., 2006), and reduced cardiac hypertrophy in spontaneous hypertensive rats (Wang et al., 2009).

Interestingly, ECM turnover was implicated in the release of TGFβ into the interstitial space, which was associated with myofibroblast conversion and enhanced collagen deposition following pressure overload-induced hypertrophy (Zile et al., 2012) and MI (Spinale et al., 2010). Besides the impact upon paracrine signaling, the activity of matrix-degrading enzymes, namely MMP-7, was implicated in proteolytic cleavage of Cx43 and electrical remodeling post-MI (Lindsey et al., 2006), reinforcing the role of the ECM as an integrative platform for cellular crosstalk.

Aiming to establish common MMPs substrates that could play a role in cardiac remodeling *in vivo*, proteomic analyses of infarcted hearts from mice lacking either MMP-7 (Chiao et al., 2010) or MMP-9 (Zamilpa et al., 2010) identified the matricellular protein tenascin-C. Interestingly, not only increased levels of tenascin-C have been found in cultured cells exposed to hypoxia or Ang II (Gonçalves et al., 2019), but also in the serum of patients with hypertensive heart disease (Franz et al., 2009), hypertrophic cardiomyopathy (Kitaoka et al., 2012) and MI (Imanaka-Yoshida et al., 2001), likely representing a valuable prognostic biomarker of HF. In the setting of MI, tenascin-C was implicated in the regulation of inflammation and ECM remodeling, by modulating the expression of MMP-9, TIMP–1 (Santer et al., 2020) and macrophage polarization *in vivo* (Kimura et al., 2019). Moreover, recombinant tenascin-C treatment was sufficient to alter energy metabolism and induce re-expression of fetal genes in cultured cardiomyoblasts (Gonçalves et al., 2019), suggesting that tenascin-C modulates cardiac remodeling via multiple cellular targets. On the other hand, tenascin-C-null mice presented exacerbated adverse remodeling and inflammation following pressure-overload (Song et al., 2017). The different genetic background of the animals used in this study, with differences in their innate immune system, was suggested to explain the observed discrepancies (Song et al., 2017), reinforcing that the selection of more suitable models is key to improve translation of preclinical findings into the clinical scenario.

## Pathological changes on communication mediated by soluble factors

In the diseased myocardium, increased TGFβ was demonstrated not only to induce gene transcription changes in fibroblasts, triggering a pro-fibrotic genetic program (Basma et al., 2019), but also to modulate the electrophysiological properties of myofibroblasts (Salvarani et al., 2017), as well as hypertrophic cardiomyocyte growth (Schultz et al., 2002). Although the molecular mechanisms remain unclear, Cx43 expression is required for TGFβ-mediated myofibroblast differentiation (Asazuma-Nakamura et al., 2009). Consistent with the hypothesis that such effects are driven by non-junctional functions of Cx43, previous studies reported that Cx43 binding to microtubules results in the release and nuclear translocation of Smad2/3, ultimately activating TGFβ signaling (Dai et al., 2007).

Secretion of other growth factors and cytokines have also been associated with cardiac remodeling. In agreement, multiple studies have implicated the proinflammatory cytokine IL-6 in the pathophysiology of left ventricle remodeling following MI (Groot et al., 2019) and pressure overload (Lai et al., 2012). Although the precise contribution of IL-6 to cardiac diseases remained controversial for several years (Fuchs et al., 2003; Lai et al., 2012), a more recent study demonstrated that IL-6 knockout prevented cardiac remodeling and dysfunction in mice subjected to transverse aortic constriction (TAC) (Zhao et al., 2016). Importantly, higher plasma levels of IL-6 were correlated with larger infarct size and decreased cardiac function in ST-elevation myocardial infarction (STEMI) patients (Groot et al., 2019). On the other hand, evidence associating IL-6 with a beneficial impact on infarct wound healing has also been reported (Mayfield et al., 2017), reinforcing the pleiotropic nature of IL-6 functions. In addition to its soluble form, IL-6 was found in EV derived from hypertrophic cardiomyocytes, likely contributing to the pro-fibrotic effects of these vesicles (Datta et al., 2017).

## Pathological changes on communication mediated by extracellular vesicles

Ample evidence has demonstrated an increased number of circulating EV in animal models of ischemia/reperfusion (Deddens et al., 2016; Martins-Marques et al., 2020a) and cardiac pressure overload (Pironti et al., 2015), which may contribute to the pathophysiology of disease. In agreement, circulating EV from human patients following MI modulate the inflammatory profile of cultured macrophages (Almeida Paiva et al., 2019), whereas circulating vesicles from MI convalescence patients ameliorate oxidative damage in endothelial cells (Cao et al., 2020).

In addition, changes in the protein and miRNA content of circulating EV have been recognized as relevant markers of disease progression and/or prognosis. For example, circulating EV in mouse models of acute MI (Cheng et al., 2019; Ling et al., 2020) and ischemia/reperfusion (Deddens et al., 2016) display higher levels of miRNA-1, miRNA-21, miRNA-126, miRNA-208, and miRNA-499, whereas miRNA-27a* (Tian et al., 2020) and miRNA-146a (Beg et al., 2017) were found upregulated during chronic HF in mice and humans, respectively. Additionally, higher levels of MMP-9 (Chen et al., 2020) and phosphatase and tensin homolog (PTEN) (Ling et al., 2020), as well as lower levels of Cx43 (Martins-Marques et al., 2020a) were identified in circulating EV from human MI patients, posing as promising circulating markers of disease.

Although the cellular origin of circulating EV remains unclear, *in vitro* studies have contributed to elucidate the signals underlying disease-induced EV secretion by specific cell populations. Consistently, *in vitro* stimulation of fibroblasts with Ang II, a potent hypertrophic factor, results in increased secretion of EV (Lyu et al., 2015). Moreover, mouse models with cardiac-specific transgenic gene expression represent promising approaches to yield further insights. Such models enabled not only the identification of cardiomyocyte-derived EV enriched in Ang II Type I Receptor (AT1R) in the circulation of mice subjected to TAC, but also that EV-mediated transfer of functional AT1R into vascular cells regulates blood pressure responses (Pironti et al., 2015). Although *in vivo* spatiotemporal tracking of EV remains a challenging task, a cardiomyocyte-specific EV-tracking mouse, based on the expression of the EV surface marker CD63 fused to a nanoluciferase reporter, was recently proposed as a powerful model to visualize endogenous EV uptake and to determine their biological effects (Luo et al., 2020).

Importantly, *in vitro* approaches have given valuable contributions to establish the biological functions of EV produced in a disease-associated milieu, namely their impact after being taken up by specific target cells. For example, EV derived from fibroblasts treated with Ang II (Lyu et al., 2015) or TGFβ (Basma et al., 2019) were able to stimulate pro-hypertrophic signaling in recipient cardiomyocytes. These effects have been related with the transfer of specific EV-enclosed miRNA, including miRNA-21*, from fibroblasts to cardiomyocytes (Bang et al., 2014). In turn, miRNA-208a-enriched EV derived from hypoxic, ischemic or Ang II-stimulated cardiomyocytes enabled fibroblast proliferation and conversion into myofibroblasts (Morelli et al., 2020; Yang et al., 2018). Similarly, EV released by ischemic cardiomyocytes elicit angiogenesis of cardiac endothelial cells, mainly due to the transfer of miRNA-143 and miRNA-222 (Ribeiro-Rodrigues et al., 2017b).

## The importance of intercellular communication in cell-based therapies

### The impact of direct cell–cell communication on cell-based therapies

Currently, the most effective therapy for STEMI patients relies on timely reperfusion by primary percutaneous intervention (PCI) or coronary artery bypass grafting (CABG) (Thygesen et al., 2019), which is vital to salvage the affected myocardium, but has also been paradoxically associated with the induction of a second wave of injury termed reperfusion injury (Heusch, 2020). Although revascularization strategies have successfully contributed to reduce overall mortality, the increasing number of surviving STEMI patients emphasizes the urgent need to develop novel strategies able to preserve myocardial function and prevent maladaptive hypertrophy, HF onset and progress.

**Fig. 3.**
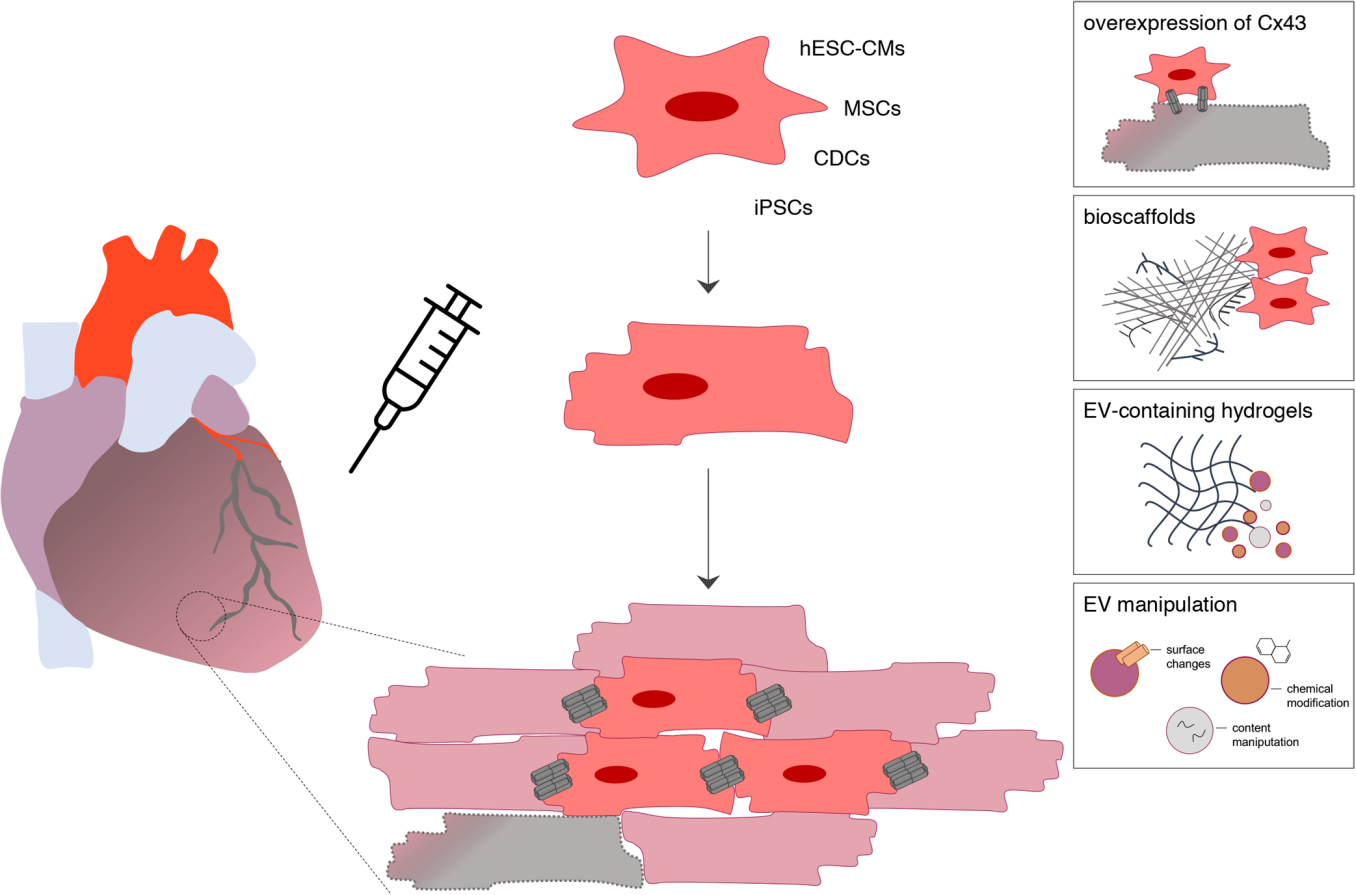
**Strategies to improve the efficacy of cell-based therapies.** Cardiac regeneration in heart failure has been attempted with the transplantation of mesenchymal stem cells (MSCs), cardiosphere-derived cells (CDCs), and cardiomyocytes derived from human embryonic stem cells (hESC-CMs) or induced-pluripotent stem cells (iPSCs). Targeting different cell–cell crosstalk pathways is emerging as an appealing strategy to improve the success of cell-based therapies. Overexpression of Cx43 in transplanted cells, the development of ECM-inspired biomaterials or hydrogels enriched in extracellular vesicles that may also be chemically or biologically manipulated, could enhance electromechanical host-graft coupling, angiogenesis and native cardiomyocyte survival, thereby potentiating its beneficial effect.

Several clinical trials, including the ALLSTAR (NCT01458405), DYNAMIC (NCT02293603), CADUCEUS (NCT00893360) and PreSERVE-AMI (NCT01495364) reported that intracoronary administration of allogeneic cardiosphere-derived cells (CDCs) or bone marrow CD34+ cells is safe (Chakravarty et al., 2021; Makkar et al., 2020; Malliaras et al., 2014; Quyyumi et al., 2017). Nonetheless, its unequivocally efficacy in improving cardiac function in MI and HF patients remains controversial. In fact, cell-based therapies display numerous challenges, including the limited engraftment and survival of transplanted cells, as well as the lack of electromechanical coupling with host injured cardiomyocytes, which likely contribute to the development of ventricular arrhythmias (Madonna et al., 2016). In this regard, targeting different cell–cell communication mechanisms is emerging as a promising strategy (Fig. 3). For example, overexpression of Cx43 in transplanted autologous skeletal myoblasts was suggested to improve electrical coupling between host and engrafted cells (Antanavičiute et al., 2015; Fernandes et al., 2009). Besides propagation of electrical signals, GJ-mediated exchange of signaling molecules may enable differentiation of transplanted mesenchymal stem cells (MSCs) into cardiac progenitors (Lemcke et al., 2017), which may also contribute to improve the success of these strategies. However, although host-graft GJ-mediated connections have been detected *in vivo* after transplantation of human embryonic stem-cell-derived cardiomyocytes (hESC-CMs), non-fatal ventricular arrhythmias were still observed in recipient non-human primate hearts (Chong et al., 2014), suggesting that other factors determine the electrical and mechanical integration of engrafted cells, namely their intrinsic excitable properties or functional maturation.

Although in the absence of *in vivo* evidence, TNT-mediated communication between transplanted cells and remote cardiomyocytes has been proposed as an alternative mechanism to ensure proper electrical coupling (Fernandes et al., 2009). Importantly, transfer of MSCs mitochondria via TNT-like connections was accompanied by adult cardiomyocyte reprograming towards a progenitor-like state, which may have relevant implications to cell survival and proliferation (Acquistapace et al., 2011). TNT-mediated transfer of functional mitochondria into injured cardiomyocytes may also enhance functional cardiac recovery following MI (Han et al., 2016).

Despite being underestimated for years, it is conceivable that an adequate interplay between transplanted cells and the host ECM is vital for the success of cellular therapies, sustaining proper transplanted cell adhesion and behavior. In fact, transplanted cells are able to affect the biomechanical properties of host tissue (Berry et al., 2006) and activate intrinsic acute inflammatory-based healing responses (Vagnozzi et al., 2020), reducing myocardial stiffness and improving cardiac function. Moreover, paracrine signals released by transplanted vascular smooth muscle cells were shown to preserve collagen content and ECM architecture in host injured hearts (Fedak et al., 2012). Nonetheless, dramatic changes in the host ECM composition and stiffness following MI may impair efficient cardiac differentiation of MSCs (Sullivan et al., 2014), which highlights the need to better understand the signals and molecular players underlying these effects.

### The impact of paracrine signaling on cell-based therapies

Given its poor engraftment, cardiac regeneration induced by cell-based therapies has been mostly associated with paracrine signals secreted by transplanted cells, as well as with the stimulation of endogenous cardiac repair mechanisms (Feyen et al., 2017; Madonna et al., 2016). Therefore, recent strategies have focused in improving the delivery of cardioprotective factors. In accordance, intrapericardial injection of hydrogels containing induced-pluripotent stem cells (iPSCs) or MSCs-derived EV was proposed as a valuable strategy to enhance cardiac repair following MI, presenting decreased inflammatory cell infiltration (Zhu et al., 2021). More recently, subcutaneously implanted stem cells-carrying devices demonstrated powerful cardioreparative properties in a mouse model of MI, which were correlated with the presence of stem-cell-derived EV in the scar, infarct border and remote regions of the myocardium 4weeks post-MI (Kompa et al., 2021). While this study reinforced the importance of systemic paracrine signals in promoting cardiac repair, its relevance as predictive markers of cardioprotective potency was also suggested, after non-invasive monitoring of plasma-based EV miRNA content of transplant recipients (Saha et al., 2019).

Importantly, the addition of a bioscaffold during cell transplantation was shown to act as a key factor to improve microenvironmental support in cell-based therapies, namely by preventing cell-detachment-induced apoptosis (anoikis) (He et al., 2015). Accordingly, transplantation of muscle patches formed by cardiomyocytes, endothelial cells and smooth muscle cells derived from human iPSCs in a fibrin scaffold improved cardiac function in a large animal model of MI (Gao et al., 2018). This strategy presented with an enhanced engraftment rate in the absence of arrhythmogenic complications, and was accompanied by increased angiogenesis and native cardiomyocyte survival, which were largely associated with EV secretion (Gao et al., 2018). In fact, strategies that synergistically target heterocellular connections and vascular regeneration through the secretion of paracrine factors presented encouraging outcomes after MI (Park et al., 2019). Besides its effects in promoting angiogenesis and ameliorating inflammation, paracrine signals from transplanted cells were reported to modulate the activity of cardiac Ca^2+^ channels, thereby indirectly affecting excitation-contraction coupling, strengthening its therapeutic potential (DeSantiago et al., 2012).

Although cell-based therapies targeting cardiac hypertrophy remain less explored, intramyocardial delivery of MSCs was shown to improve neovascularization and reduce cardiomyocyte hypertrophy, ameliorating cardiac remodeling in animal models of right ventricle pressure and volume overload (Liufu et al., 2020; Wehman et al., 2016; Yerebakan et al., 2009).

## Concluding remarks and future perspectives

Major alterations in cell–cell crosstalk have been associated with the onset and progression of several cardiac disorders, including myocardial infarction and cardiac hypertrophy. Therefore, improving our understanding on the regulatory mechanisms underlying intercellular communication in health and disease conditions, is instrumental not only to identify novel molecular targets, but also to enhance the efficacy of current therapies. Despite being often studied as independent mechanisms, a major future challenge lies in finding more appropriate models to study the different intercellular communication pathways in an integrated manner, either *in silico*, *in vivo* or resorting to innovative 3D heart-on chip models. For example, while for decades, arrhythmogenesis has been associated with dysfunctional GJIC, TNT-mediated communication was revealed to increase under several pathological scenarios. Given the importance of TNT in electrical transmission, it will be relevant to establish whether TNT-mediated communication can be activated as a compensatory mechanism in the diseased heart, ensuring electric and/or metabolic coupling across scar tissue. As TNT participate both in the transfer of cardioprotective and pro-apoptotic signals, it is critical to elucidate its exact role in the context of cardiac ischemia and hypertrophy, which would greatly benefit from the development of more reliable models. Moreover, uncovering strategies that selectively enable electrical synchrony, either via GJ or TNT, but restrain the propagation of harmful signals from injured to healthy myocardial areas will be vital.

Although underestimated for years, the emerging concept that non-excitable cells also play essential roles upon cardiac pathophysiology, through direct electrical coupling or EV-mediated paracrine signaling, requires further investigation. Connecting all cardiac cell populations, the ECM that is subjected to major remodeling in the diseased heart, will certainly constitute a hot topic in future research. Particularly, it will be important to establish to what extent ECM remodeling interferes with the release of matrix-bound growth factors and EV, as well as with TNT formation and Cx43 levels, ultimately affecting intercellular communication at distinct levels. In this setting, computational simulation and machine learning tools are expected to significantly contribute to dissect reciprocal regulation mechanisms involving distinct cell–cell crosstalk pathways and multiple cell types (Mayourian et al., 2017).

Although with very promising results in preclinical models, many cardioprotective and regenerative therapies have shown disappointing outcomes in the clinical setting. Recent evidence demonstrated that targeting cell–cell communication mechanisms could improve the success of cell-based therapies, providing enhanced cellular engraftment, and stimulating endogenous wound healing responses, in which the importance of the ECM has been increasingly recognized. In fact, ECM-inspired bioscaffolds promote cardiac regeneration, by conferring structural support and acting as a dynamic reservoir of biomolecules that elicit angiogenesis and inflammation. In this regard, a better characterization of the impact of soluble factors and EV in the injured myocardium will be key to improve these strategies. Leveraged by the development of more sophisticated techniques, the rapidly expanding field of EV biology is expected to provide deeper insights into the EV biogenesis and uptake mechanisms. Definitely, the identification of specific markers that render the purification of more homogeneous bioactive EV subsets, devoid of contaminants, will contribute not only to propel the design of novel cardioreparative strategies, but also to serve as relevant markers of prognosis and response to therapy. Major roadblocks to clinical translation of EV-based therapeutics lie in the lack of standardized, scalable and reproducible isolation procedures, as well as in the development of strategies able to direct EV into specific cell populations, which warrant future investigation.

## Funding
